# Nanoengineered Cobalt Electrocatalyst for Alkaline Oxygen Evolution Reaction

**DOI:** 10.3390/nano14110946

**Published:** 2024-05-28

**Authors:** Venkatachalam Rajagopal, Sunil Mehla, Lathe A. Jones, Suresh K. Bhargava

**Affiliations:** Centre for Advanced Materials and Industrial Chemistry (CAMIC), School of Science, STEM College, RMIT University, GPO Box 2476, Melbourne, VIC 3001, Australia; rajagopal.venkatachalam@rmit.edu.au (V.R.); sunil.mehla@rmit.edu.au (S.M.);

**Keywords:** cobalt, hydrogen bubble templating, electrocatalyst, oxygen evolution reaction, water electrolysis

## Abstract

The alkaline oxygen evolution reaction (OER) remains a bottleneck in green hydrogen production owing to its slow reaction kinetics and low catalytic efficiencies of earth abundant electrocatalysts in the alkaline OER reaction. This study investigates the OER performance of hierarchically porous cobalt electrocatalysts synthesized using the dynamic hydrogen bubble templating (DHBT) method. Characterization studies revealed that electrocatalysts synthesized under optimized conditions using the DHBT method consisted of cobalt nanosheets, and hierarchical porosity with macropores distributed in a honeycomb network and mesopores distributed between cobalt nanosheets. Moreover, X-ray photoelectron spectroscopy studies revealed the presence of Co(OH)_2_ as the predominant surface cobalt species while Raman studies revealed the presence of the cubic Co_3_O_4_ phase in the synthesized electrocatalysts. The best performing electrocatalyst required only 360 mV of overpotential to initiate a current density of 10 mA cm^−2^, exhibited a Tafel slope of 37 mV dec^−1^, and stable OER activity over 24 h. The DHBT method offers a facile, low cost and rapid synthesis approach for preparation for highly efficient cobalt electrocatalysts.

## 1. Introduction

Availability of green hydrogen at low cost is critical for the transition away from a fossil fuel economy towards a circular carbon economy and a clean energy abundant society [1,2]. Electrocatalytic water splitting is one of the most promising technologies for the production of green hydrogen owing to the abundant supply of water and renewable energy sources on earth, and the high efficiency and safety of the water electrolysis process [3,4]. According to the International Energy Agency’s world energy outlook report, the market share of water electrolysis in green hydrogen production is anticipated to reach 22% by 2050 [5]. Moreover, water electrolysis will also play an important role in emerging short-term and long-term energy storage solutions essentially addressing both seasonal and day to day energy demands by storing energy in the chemical form as water [6]. However, the sluggish reaction kinetics of the oxygen evolution reaction (OER) and the poor stability of electrocatalysts in alkaline water splitting are major bottlenecks which seriously affect the economic viability and rapid commercial uptake of this promising technology [7,8,9,10,11].

The kinetics of OER is sluggish because it involves multi-proton coupling, multi-electron transfer, and formation of O-O bonds in four redox processes [12]. The requirement of a high overpotential in OER is related to the energy required to overcome this kinetic barrier [10]. Ruthenium dioxide (RuO_2_) with a rutile structure exhibits excellent OER activity in both alkaline and acidic mediums [13]. However, RuO_2_ is unstable during OER in both mediums, oxidizes to RuO_4_ and dissolves in the aqueous electrolyte [14]. Iridium dioxide (IrO_2_) is more stable than RuO_2_ and can sustain higher anodic potential in both mediums [15,16]. However, IrO_2_ oxidizes to IrO_3_ and is consumed in the OER through dissolution [17]. The high costs of these promising noble metal electrocatalysts, their scarcity and poor stability in alkaline OER has fuelled the search for cost-effective and earth-abundant electrocatalysts with high OER activities. As an earth abundant metal, cobalt-based electrocatalysts have recently received wide attention owing to their high electrocatalytic activities and stable performance in the alkaline medium [18,19]. The strong oxophilicity of cobalt accelerates water dissociation before the adsorption of H_ad_ through the Volmer step [18]. It also results in strong adsorption of hydroxyl ions on the surface of cobalt-based electrocatalysts. Different types of cobalt-based electrocatalysts investigated for OER include CoO, Co_3_O_4_, Co_4_O_4_ cubane, cobalt hydroxide, cobalt oxyhydroxide, perovskite, cobalt phosphide, cobalt borate, and cobalt sulfide [7,20,21,22,23]. Besides optimization of the composition of cobalt-based electrocatalysts, their stability during OER is an area of active investigation. Cobalt electrocatalysts often exhibit mixed valence states of Co^2+/3+/4+^ in the different constituent phases but these phases are known to transform to hydroxides and oxyhydroxides during the oxygen evolution reaction [9,24,25]. For instance, Co_2_P [26] and CoF_2_ electrocatalysts rapidly transform to Co(OH)_2_ under the reaction conditions. Budiyanto et al. recently confirmed the excellent morphological stability and OER activity of mesostructured Co_3_O_4_ electrocatalysts in commercially relevant highly alkaline solutions (13 M KOH) over extended durations up to 12 h [27]. In the acidic medium, however, cobalt oxides rapidly dissolve leading to a collapse of the crystal structure and loss of OER activity [28,29].

Other important characteristics which significantly influence OER activity include the morphology, porosity, and charge transfer resistance of the prepared electrocatalysts. The same material when prepared in different morphologies exposes different types of crystal planes to the reaction intermediates which influences the catalytic activity [30]. Porosity on the other hand governs the adsorption and diffusion of the reactant intermediates towards the active sites and diffusion of the product intermediates away from the active sites. An ideal electrocatalyst should have a uniform dispersion of the active sites throughout its surface and these active sites should be easily accessible through uniform pores. In water electrolysis, particle morphology and porosity also govern the formation and separation of hydrogen and oxygen bubbles from the electrodes which in turn affects the overpotential and overall catalytic activity [31,32]. Finally, the charge transfer resistance of the prepared electrocatalyst is also important since it governs the adsorption transfer resistance of the hydroxyl (OH*) intermediates [33]. The unique mechanical and chemical properties, high catalytic activities and high specific surface areas of porous materials has also led to their wide adoption in batteries, supercapacitors, catalysis, sensors, and fuel cell applications. Porous electrocatalysts can be prepared using a variety of methods including the use of hydrothermal and electrodeposition methods [34], use of close-packed colloidal crystals as templates [35,36], lithography methods [37], lyotropic liquid crystalline phases of surfactants as templates [38], or by using bio-templates such as sea urchin skeletal plates [39].

**Scheme 1:**
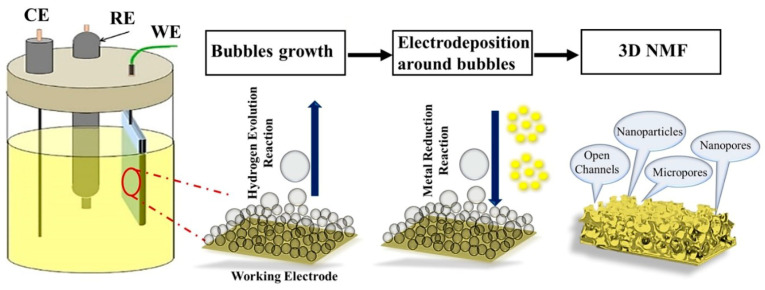
Synthesis of porous materials using dynamic hydrogen bubble templating. Adapted with permission from [44] Copyright© 2022 John Wiley and Sons.

Recently, a rapid, facile, and environmentally benign synthesis technique, termed the dynamic hydrogen bubble templating (DHBT) method was proposed for the synthesis of ordered macroporous materials. This method employed hydrogen bubbles evolved during electrodeposition as a dynamic template for imbibing porosity to the synthesized materials as illustrated in Scheme 1 [40,41,42,43,44]. The evolution of hydrogen bubbles during electrodeposition occurred from the electrolysis of the used aqueous electrolytes. Tsai et al. used phase-contrast radiology with synchrotron radiation to confirm that the in situ generated hydrogen bubbles acted as a physical template to direct the electrodeposited nanostructures into porous honeycomb networks with formation of near-spherical and rod-like pores [45]. The DHBT method offers several advantages such as low cost, ease of operation, good control over the porous structure, distribution of pores in a honeycomb-like network, and no requirement for the removal of template materials after deposition [43]. In our group we have previously demonstrated the use of the DHBT method to synthesize copper-gold alloys for the reduction of 4-nitrophenol, porous platinum for methanol oxidation [46], porous copper-palladium alloys for hydrogen evolution reaction [47], and other nanostructured electrocatalysts for various applications [48,49]. This study focuses on the synthesis of hierarchically porous cobalt electrocatalysts and investigation of their performance in the alkaline oxygen evolution reaction.

## 2. Materials and Methods

### 2.1. Chemicals

Cobalt (II) chloride hexahydrate (CoCl_2_·6H_2_O, 98%), ammonium chloride (NH_4_Cl, 99.5%), polyethylene glycol (PEG, 99%), sodium hydroxide (NaOH, 98%), ruthenium (IV) oxide (RuO_2_, 99.9%), and copper plates were procured from Sigma Aldrich (Burlington, MA, USA). Milli-Q water (Rahway, NJ, USA) (18.2 M·cm) was used for all synthesis, cleaning, and testing procedures.

### 2.2. DHBT Synthesis of Hierarchically Porous Cobalt Electrocatalysts

Copper plates were sheared into rectangular strips of dimension 4 cm × 1 cm. A conductive area of 1 cm × 1 cm was left exposed on these copper substrates by masking the edges, back side, and 3 cm length on the front side of these strips with Kapton (polyimide) tape. The copper substrates were cleaned with deionised water, ethanol, and isopropanol prior to electrodeposition. Electrodeposition was performed in the two-electrode configuration at room temperature with a graphite rod (9 cm^2^) as the counter electrode and the masked copper strips as the working electrode. The electrolyte was prepared by dissolving 0.12 M CoCl_2_·6H_2_O, 1.5 M NH_4_Cl and 0.01 M PEG in 10 mL deionized water. Electrodeposition was allowed to complete for time intervals of 10 s (Co@Cu-10), 20 s (Co@Cu-20), and 30 s (Co@Cu-30) at a constant current density of 3 A/cm^2^. The electrodeposition of a porous cobalt film could be visually observed as a matt black film on the copper substrates. All samples were first examined under an optical microscope to check the presence of macropores before further investigation with a scanning electron microscope. The electrochemical activities of the synthesized samples were compared against a RuO_2_ reference electrode. The reference electrode was prepared by mixing 10 mg of commercial RuO_2_ powder in a mixture of ethanol (50%) and water (50%), with the addition of a few drops of 5% Nafion solution and ultrasonication for 10 min. This slurry was drop casted on the copper substrate followed by drying to obtain the reference RuO_2_ electrode.

### 2.3. Oxygen Evolution Reaction Measurements

The electrochemical analysis for the OER was carried out using a CH Instruments (Austin, TX, USA) (CHI 760C) electrochemical analyzer equipped with a standard three-electrode system.

The working electrode consisted of honeycomb Co@Cu electrodes deposited for different durations (10 s, 20 s, and 30 s). A platinum wire served as the auxiliary electrode, and an Ag/AgCl (3 M KCl) electrode was used as the reference electrode. Linear sweep voltammetry (LSV), electrochemical impedance spectroscopy (EIS), and chronoamperometry (CA) for the synthesized electrodes were performed in 1 M NaOH solution.

## 3. Results and Discussion

**Figure 1:**
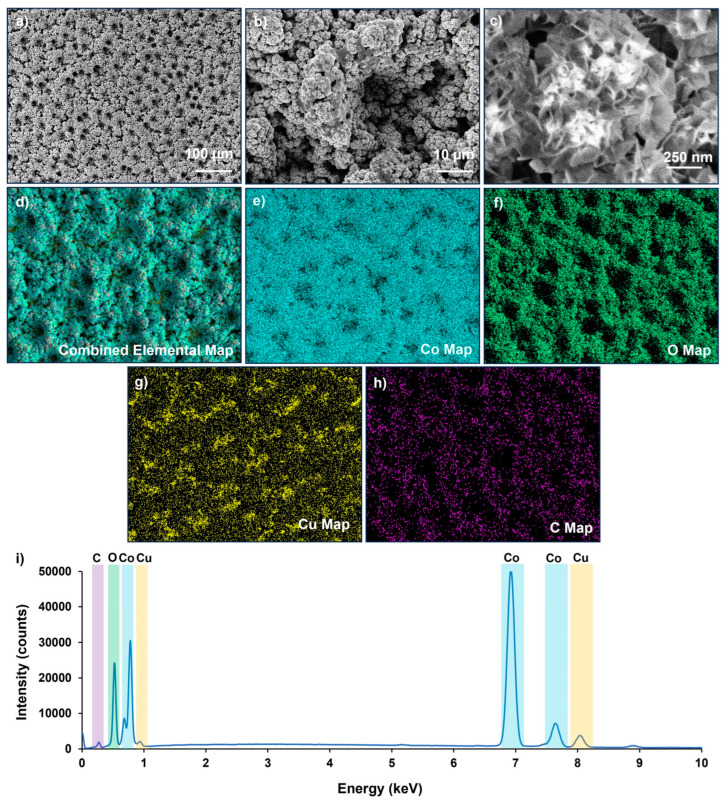
Scanning electron microscope (SEM) and energy dispersive X-ray (EDX) analysis of the cobalt films electrodeposited using the dynamic hydrogen bubble templating method for 30 s. SEM images at (**a**) low magnification, (**b**) medium magnification, (**c**) high magnification, (**d**) combined EDX elemental map, (**e**) cobalt map, (**f**) oxygen map, (**g**) copper map, (**h**) carbon map, and (**i**) the EDX spectrum obtained from the sample.

The porous cobalt films electrodeposited for time intervals of 10, 20 and 30 s were characterized using FEI Quanta 200 and FEI Verios 460 L (FEI Company, Hillsboro, OR, USA) extra high resolution (XHR) scanning electron microscopes (SEM). The SEM images of the different samples at low and high magnifications are depicted in Figure S1. The low magnification SEM images (S1a, S1b and S1c) indicated the presence of a continuous cobalt film with spherical macropores distributed in a honeycomb-like network in all the samples. These circular macropores were generated due to the evolution of hydrogen bubbles during electrodeposition which essentially acted like a dielectric template to direct the structure of electrodeposited material. The diameter of these spherical macropores roughly varied from 10 to 20 µm. High magnification SEM images of the material electrodeposited around the macropores (Figure S1d–f) confirmed the presence of mesoporosity (pores with diameters between 2 and 50 nm) in the electrodeposited structures. The diameter of these mesopores as estimated from SEM images roughly varied between 20 and 50 nm. Interestingly, the cobalt film which was obtained from electrodeposition for a time interval of 30 s (Co@Cu-30) consisted of well-defined nanosheets which were vertically aligned in a flower-like morphology. The XHR SEM images for the Co@Cu-30 sample at three different magnifications are depicted in Figure 1a–c. SEM imaging confirmed that Co@Cu-30 electrocatalyst consisted of hierarchical porosity i.e., both macropores (10–20 µm) and mesopores (20–50 nm), and vertically aligned cobalt nanosheets. The mesopores were also present in Co@Cu-10 and Co@Cu-20 electrocatalysts but with the absence of any well-defined cobalt nanosheets as evident from Figure S1.

The energy dispersive X-ray (EDX) analysis for Co@Cu-30 electrocatalyst confirmed the presence of Co, O, Cu, and C as evident from Figure 1c–i. The Co@Cu-10 and Co@Cu-20 samples also exhibited a similar elemental composition which is summarized in Table 1. The copper peaks originated from the copper substrate while the carbon peaks originated from the trace amounts of PEG adsorbed on the surface of electrocatalysts. The oxygen peaks in the EDS spectra confirmed the presence of some oxidized cobalt phases in the electrodeposited samples. These oxidized phases mainly consisted of cobalt hydroxide (Co(OH)_2_) on the surface confirmed through XPS studies and cobalt (II,III) oxide (Co_3_O_4_) in bulk confirmed through Raman spectroscopy. From Table 1 it was clear that the Co@Cu-30 electrocatalyst had the highest fraction of cobalt content in the three samples, and the lowest oxygen content. The increase in cobalt content with electrodeposition duration was related to the increase in thickness of the deposited films. The decrease in oxygen content with the increase in electrodeposition duration supported the conclusion that the oxidized cobalt species, Co(OH)_2_ and Co_2_O_3_, were only present near the surface of the synthesized electrocatalysts while the bulk of the electrocatalyst mainly contained metallic cobalt.

**Figure 2:**
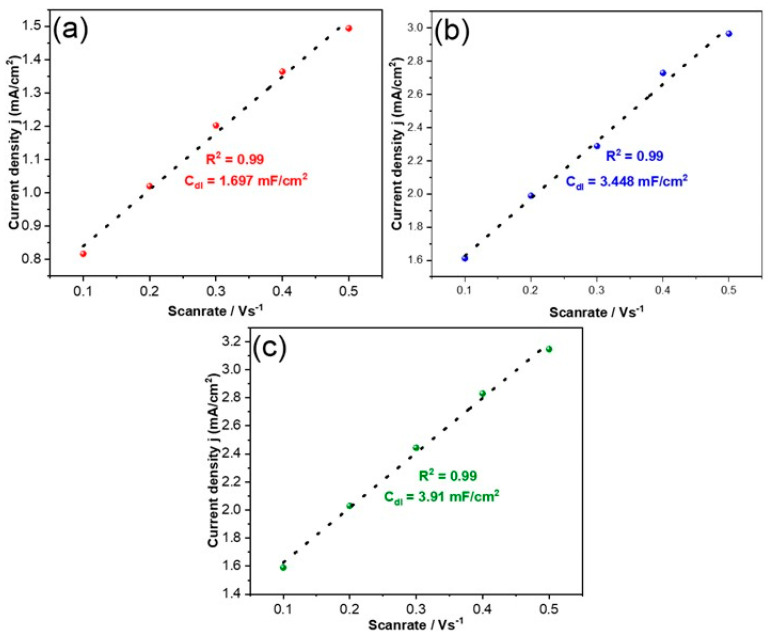
Determination of the electrochemical double layer capacitance using linear correlation plots between non-Faradaic current density and scan rates for (**a**) Co@Cu-10, (**b**) Co@Cu-20, and (**c**) Co@Cu-30.

The electrochemical double-layer capacitance (C_dl_) for each sample was calculated using the method described elsewhere [50]. In short, the capacitance was determined from scan rate dependent cyclic voltammograms (CVs) by estimation of the non-Faradaic capacitive current associated with double-layer charging. A potential window, typically within 0.1 V centred at the open-circuit potential (OCP) of the system, was first identified from the static cyclic voltammogram which was devoid of any apparent Faradaic processes. Within this non-Faradaic potential region, all measured current density was attributed to double-layer charging. The double-layer capacitance (C_dl_) for each sample was obtained from the slopes of a linear plot between current density and scan rate as depicted in Figure 2a–c, respectively. The electrochemical double layer capacitance for the different samples increased in the following order: 1.697 mF/cm^2^ (Co@Cu-10) < 3.448 mF/cm^2^ (Co@Cu-20) < 3.91 mF/cm^2^ (Co@Cu-30). The electrochemical double layer capacitance is often correlated to the electrochemically active surface area of electrocatalysts [51]. The observed trend confirmed that the porosity of the cobalt electrocatalysts and their electrochemically active surface areas increased with an increase in electrodeposition duration with the Co@Cu-30 electrocatalyst exhibiting the highest value.

**Figure 3:**
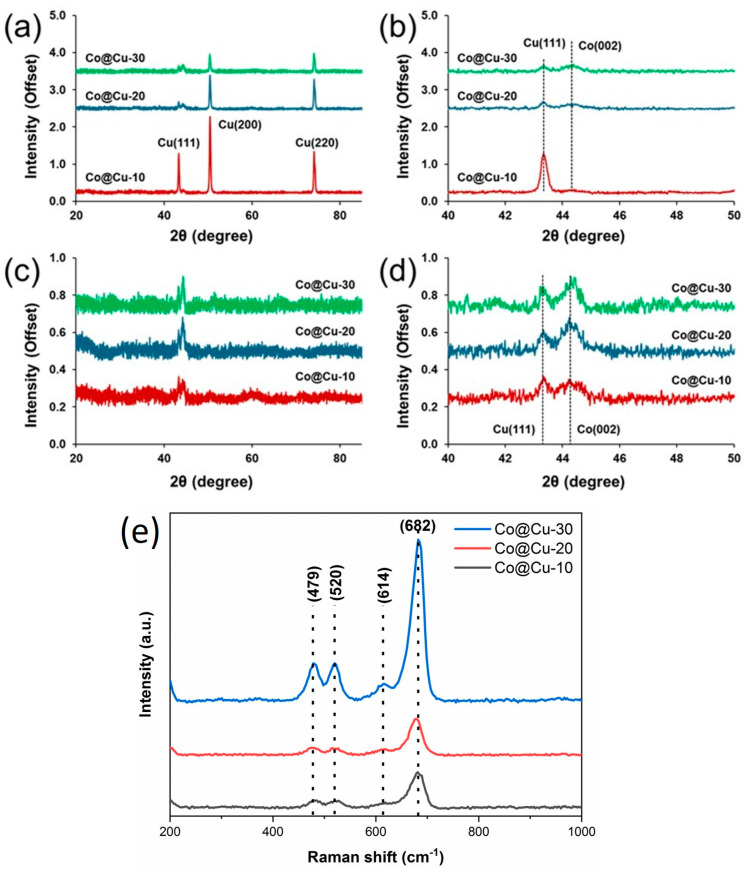
X-ray diffractograms for DHBT-synthesized cobalt electrocatalysts on different substrates (**a**) on copper substrates in the 2θ range from 20 to 85 degrees, (**b**) on copper substrates in the 2θ range from 40 to 50 degrees, (**c**) on glass substrates in the 2θ range from 20 to 85 degrees, (**d**) on glass substrates in the 2θ range from 40 to 50 degrees, and (**e**) Raman spectra of cobalt electrocatalysts collected using a 532 nm laser.

The DHBT-synthesized cobalt electrocatalysts were further characterized by X-ray diffraction (XRD) studies using a Bruker Axs D8 Discover equipment (Bruker, Billerica, MA, USA) with GADDS micro XRD detector (Bruker, Billerica, MA, USA) as described in the Supplementary Materials. The synthesized cobalt electrocatalysts were also scraped from the substrate and drop cast on glass slides for XRD analysis. The recorded diffraction patterns for the as-synthesized cobalt electrocatalysts on copper substrates and drop cast cobalt electrocatalysts on glass substrates are depicted in Figure 3. For the DHBT-synthesized cobalt electrocatalysts on copper substrates, characteristic peaks were observed at 43.5°, 50.6°, and 74.4° in all the samples which corresponded to the (111), (200), and (220) crystal facets of copper in the face-centred cubic (fcc) structure (JCPDS #85–1326), respectively [52]. These copper peaks originated from the copper substrates. Moreover, all electrocatalysts exhibited a broad peak at 44.74° which corresponded to the (002) crystal facets of cobalt in a hexagonally closed packed (hcp) arrangement (JCPDS 05-0727), respectively [53]. Figure 3b depicts the recorded diffraction patterns for all samples selectively in the 2θ range from 40 to 50° where the cobalt peak at 44.74° is clearly visible. The intensity of the Cu (111) peak at 43.5° was highest for Co@Cu-10 and significantly lower for Co@Cu-20 and Co@Cu-30 samples. This resulted from the higher thickness of the electrodeposited cobalt films in samples Co@Cu-20 and Co@Cu-30 and their higher cobalt content (Table 1). The contribution from copper substrates was suppressed for samples with higher cobalt content. Scraping of the porous cobalt powders from the copper substrates, their transfer to glass slides and subsequent XRD analysis led to the same conclusions. It was clear from Figure 3c,d that electrodeposited films mainly consisted of metallic cobalt with Co (002) as the most abundant cobalt crystal facets. A peak corresponding to Cu (111) was also observed for all cobalt samples on glass substrates. This was assigned to the presence of copper particles in these samples which could have resulted from the physical scraping used in preparation of these samples. Most importantly, no type of cobalt hydroxide, oxy-hydroxide, or oxide species were observed in the XRD diffractograms despite the detection of oxygen in synthesized electrocatalysts in the EDX analysis. This occurred because the detection limit of the XRD technique is limited, and it can neither detect constituents in trace amounts nor the composition of surface species. To further investigate the phase composition, synthesized cobalt electrocatalyst were characterized using a Raman spectrometer using a 532 nm laser and the collected data is shown in Figure 3e. Raman characterization confirmed the presence of cubic phase Co_3_O_4_ in all the electrodeposited samples. The four peaks identified in the Raman spectra at 479, 520, 614, and 682 cm^−1^ were assigned to the Raman active modes of E_g_, F_2g_, F_2g_, and A_1g_ for cubic Co_3_O_4_ in agreement with the previously published literature [54,55,56]. The highest Raman intensities were observed for Co@Cu-30 which had the highest cobalt content and the lowest oxygen content as determined from the EDX analysis.

**Figure 4:**
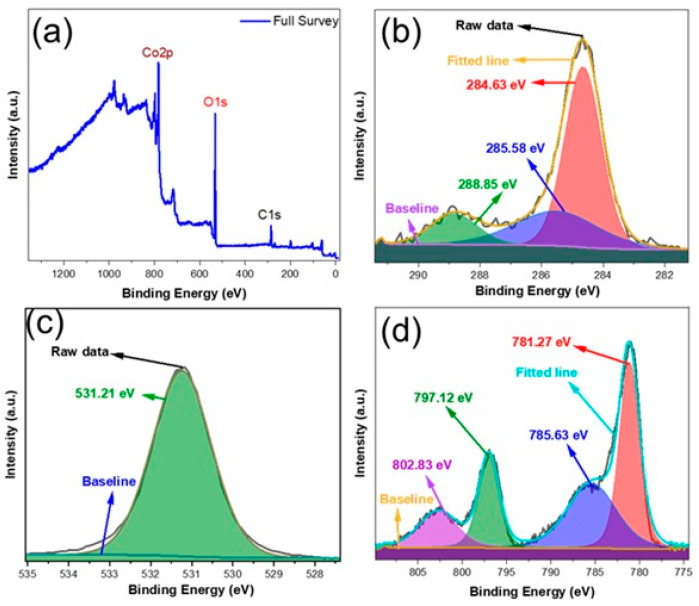
XPS characterization of Co@Cu-30 (**a**) Survey spectra, (**b**) C 1s, (**c**) O 1s, and (**d**) Co 2p spectra.

To characterize the surface of DHBT-synthesized porous cobalt electrocatalysts, all samples were further analysed by X-ray photoelectron spectroscopy (XPS) using a Thermo Scientific K-alpha XPS equipment (Thermo Fisher Scientific, Waltham, MA, USA) and the data collected are depicted in Figure 4, Figures S2 and S3, and Table S1. The XPS studies revealed the presence of only Co^+2^, C, and O (Figure 4a) in all the samples which provided the evidence that the surface of the electrodeposited catalysts mainly consisted of cobalt in an oxidized form. The carbon (C 1s) spectra obtained from Co@Cu-30 electrocatalyst as depicted in Figure 4b exhibited three distinct peaks at binding energies of 284.63, 285.58, and 288.85 eV, corresponding to the C=C, C-C, and C-O-C bonds, respectively [57]. The detection of the different types of carbon species containing C-O bonds in the XPS studies was assigned to the presence of trace levels of polyethylene glycol adsorbed on the surface of cobalt electrocatalysts. The oxygen (O 1s) spectra obtained from Co@Cu-30 as depicted in Figure 4c exhibited a single peak at a binding energy of 531.21 eV which originated from hydroxyl groups [58]. The absence of any peaks in the O 1s spectra at binding energies of 533.1 eV (surface adsorbed water) and 529.9 eV (cobalt II/III oxides) supported the conclusion that the surface of cobalt electrocatalysts predominantly consisted of cobalt in the hydroxide form [58,59]. It is well known that the surface of reactive metals gets easily oxidized on air exposure to form a fine layer of metal oxides or hydroxides. This is especially enhanced for oxophilic metals like cobalt. For instance, using monochromated XPS studies conducted at IBM research laboratory, Brundle et al. identified that exposure of polycrystalline cobalt surfaces to air at ambient conditions oxidized their surface to Co(OH)_2_ [60]. The O 1s and Co 2p spectra recorded for the DHBT-synthesized cobalt electrocatalysts in this study matched with the data reported by Bundle et al. Thus, it was concluded from the XPS studies that a thin layer of Co(OH)_2_ covered the surface of the cobalt nanosheets which could have originated from surface reconstruction under oxidative conditions during the DHBT synthesis which has been reported previously and frequently observed for cobalt-based electrocatalysts [43]. The cobalt (Co 2p) spectra obtained from Co@Cu-30 as depicted in Figure 4d exhibited four distinct peaks. The XPS peak at binding energy of 781.27 eV corresponds to the contribution of Co^+2^ 2p_3/2_ with its satellite peak at 785.63 eV [61]. The XPS peak at binding energy of 797.12 eV corresponds to the contribution of Co^+2^ 2p_1/2_ with its satellite peak at 802.83 eV [61]. The absence of any XPS peaks at binding energies of 778.4 eV and 793.3 eV confirmed the absence of any Co^+3^ species in the electrodeposited samples [61]. Similarly, peaks corresponding to metallic cobalt (777.88 eV and 800.29 eV) [57] were not observed in the XPS spectra which indicated that the surface of electrodeposited cobalt materials was completely oxidised to a hydroxide phase while the bulk of the electrodeposited films mainly consisted of Co_3_O_4_ and metallic cobalt. The samples Co@Cu-10 and Co@Cu-20 also exhibited similar features in the XPS studies as evident from Figures S2 and S3. The relative abundance of different species confirmed in the XPS studies as calculated from peak areas are depicted in Table S1. Thus, based on detailed surface characterization studies with XPS and SEM-EDX and bulk characterization with XRD and Raman spectroscopy, it becomes evident that cobalt films electrodeposited using the DHBT method consisted of macropores distributed in a near-hexagonal pattern, numerous mesopores between cobalt nanosheets, and metallic cobalt in bulk along with cubic Co_3_O_4_ while the surface of the electrodeposited films mainly consisted of Co(OH)_2_. The formation of cobalt hydroxide on the surface occurred due to the high oxophilicity of cobalt and surface reconstruction during the DHBT synthesis. The active sites for oxygen evolution reaction in the synthesized electrocatalysts were identified to be Co(OH)_2_ surface species and cubic Co_3_O_4_ in agreement with previous reports [24,55,56].

**Figure 5:**
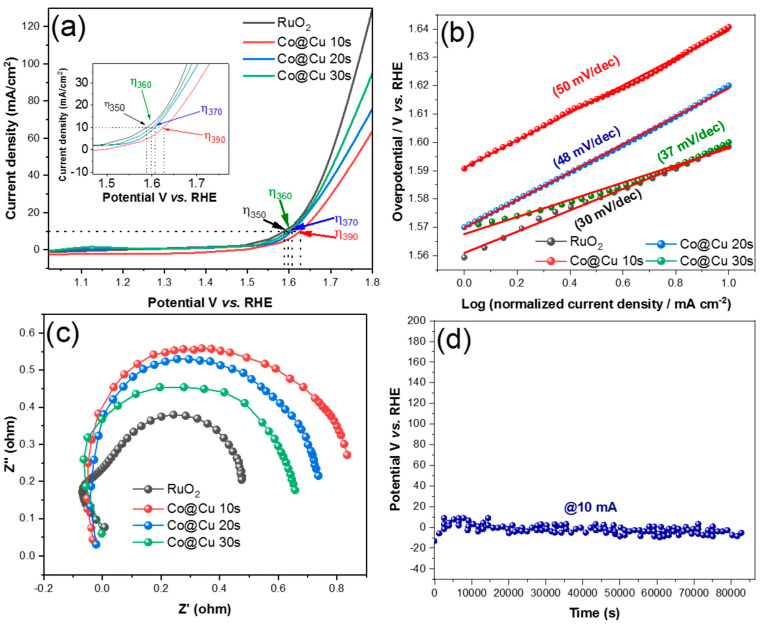
(**a**) Comparison of the electrocatalytic activity of Co@Cu-10, Co@Cu-20, Co@Cu-30 and commercial RuO_2_ in 1 M NaOH solution using linear sweep voltammetry, (**b**) Tafel slopes analysis, (**c**) AC impedance spectroscopy, and (**d**) the stability study conducted for the Co@Cu-30 electrocatalyst at the current density of 10 mA cm^−2^.

The results of the linear sweep voltammetry (LSV) studies for the DHBT-synthesized cobalt electrocatalysts and a RuO_2_ reference electrode are depicted in Figure 5a. The current increases with the increase in applied potential which originates from electrocatalytic oxygen evolution. The overpotentials for the different electrocatalysts measured at a current density of 10 mA cm^−2^ against an Ag/AgCl reference electrode (1.23 V) increased in the following order: RuO_2_ (350 mV) < Co@Cu-30 (360 mV) < Co@Cu-20 (370 mV) < Co@Cu-10 (390 mV). Thus, the performance Co@Cu-30 electrocatalyst was comparable with the refence RuO_2_ electrocatalyst. Tafel slopes are often used as a tool to compare the intrinsic catalytic activity of electrocatalysts [62]. The Tafel analysis for the electrodeposited samples which involved comparison of the sensitivity of electric current response to changes in the applied potential is depicted in Figure 5b. The Tafel slopes increased in the same order as the measured overpotentials as follows: RuO_2_ (30 mV/dec) < Co@Cu-30 (37 mV/dec) < Co@Cu-20 (48 mV/dec) < Co@Cu-10 (50 mV/dec). Again, the performance of the Co@Cu-30 electrocatalyst was close to the reference RuO_2_ catalyst while Co@Cu-20 and Co@Cu-10 electrocatalysts exhibited relatively lower intrinsic OER activity.

The charge transfer kinetics and resistance characteristics of the synthesized electrocatalysts were evaluated using AC impedance spectroscopy [63] and the results are depicted in Figure 5c. The measured impedance for the different samples increased in the following order: RuO_2_ (0.47 ohm) < Co@Cu-30 (0.65 ohm) < Co@Cu-20 (0.73 ohm) < Co@Cu-10 (0.83 ohm). These results clearly highlighted that electrocatalyst impedance could be inversely correlated with their OER activities and the RuO_2_ reference electrode exhibited the lowest impedance and the highest OER activity. The lower impedance was indicative of superior charge transfer kinetics and lower resistance to electrochemical processes in the reference RuO_2_ electrode. Comparatively, all DHBT-synthesized cobalt electrocatalysts exhibited higher impedance values with the least impedance values exhibited by Co@Cu-30. The electrical properties of the DHBT-synthesized cobalt electrocatalysts were governed by the thickness of these films and the fraction of metallic cobalt content in their bulk. The characterization studies revealed that the surface of the DHBT-synthesized cobalt electrocatalysts were completely covered with Co(OH)_2_ while the bulk contained Co_3_O_4_ in the cubic phase which were assigned as the active sites for the OER reaction in agreement with previous studies [24,55,56]. The role of metallic cobalt present in the bulk of the DHBT-synthesized electrocatalysts was to facilitate electron transport and act as a current collector. The Co@Cu-30 electrocatalyst which had the highest cobalt content exhibited lower impedance due to the presence of an efficient conducting underlayer compared to other electrocatalysts. The long-time stability test for Co@Cu-30 electrocatalyst at 10 mA cm^−2^ for 24 h is depicted in Figure 5d. This stability test clearly indicated that the potential of the Co@Cu-30 electrocatalyst and its electrocatalytic activity was stable throughout the test.

**Figure 6:**
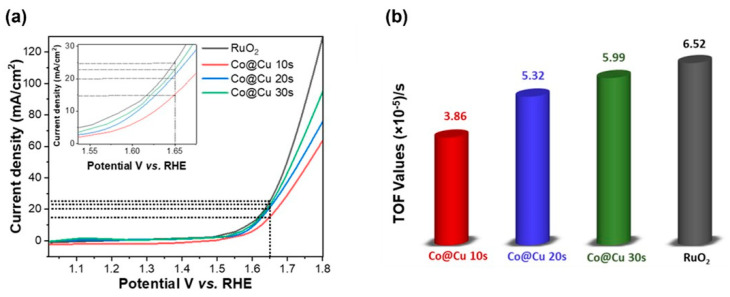
(**a**) Determination of current densities at a constant potential of 1.65 V vs. RHE in 1 M NaOH solution, and (**b**) turnover frequencies.

To compare the intrinsic reaction kinetics, the turnover frequencies (TOF) were calculated from current densities determined at a constant potential of 1.65 V vs. reversible hydrogen electrode in 1 M NaOH solution using the equation described on page 4 of the Supplementary Materials [64]. The turnover frequencies increased in the following order: RuO_2_ (6.52 × 10^−5^ s^−1^) < Co@Cu-30 (5.99 × 10^−5^ s^−1^) < Co@Cu-20 (5.32 × 10^−5^ s^−1^) < Co@Cu-10 (3.86 × 10^−5^ s^−1^) as shown in Figure 6. These results clearly highlight the significantly higher electrocatalytic activity of Co@Cu-30 compared to Co@Cu-10 and Co@Cu-20 electrocatalysts. While the presence of macropores distributed in a honeycomb-like network was a common feature of all the electrodeposited catalysts, the presence of cobalt nanosheets in Co@Cu-30, its high electrochemically active surface area (C_dl_), superior impedance characteristics, and high cobalt content were responsible for its superior electrocatalytic activity. The hierarchical porosity observed in Co@Cu-30 allowed easy diffusion of hydroxide anions to the active sites and facile transport of the evolved oxygen away from the electrodes. The higher cobalt content of Co@Cu-30 electrocatalyst contributed towards the lower impedance and better charge transfer characteristic of this electrocatalyst. The performance of hierarchically porous cobalt electrocatalyst (Co@Cu-30) synthesized in this research was benchmarked against other cobalt electrocatalysts reported in the literature for OER as shown in Table S2. It was clear from this comparison that the porous cobalt nanosheets exhibited a lower overpotential compared to some reported cobalt oxide, phosphide, sulphide, and selenide electrocatalysts.

## 4. Conclusions

The dynamic hydrogen bubble templating method employing an aqueous ammonium chloride electrolyte was successfully applied for the synthesis of a hierarchically porous cobalt electrocatalyst, Co@Cu-30, which contained near hemispherical macropores distributed in a honeycomb-like network, numerous mesopores distributed between vertically aligned cobalt nanosheets, metallic cobalt with cubic phase Co_3_O_4_ in bulk and a thin layer of Co(OH)_2_ on the surface. The superior mass and charge transfer characteristics of Co@Cu-30 along with low impedance allowed this electrocatalyst to initiate a current density of 10 mA cm^−2^ at 360 mV of overpotential, exhibit a Tafel slope of 37 mV/dec, and stable oxygen evolution electrocatalytic activity over 24 h which was comparable to a reference RuO_2_ electrocatalyst. This study provides valuable insights into the design and nanoengineering of efficient electrocatalysts for renewable energy technologies, offering a foundation for further research in the field.

## Figures and Tables

**Table 1:** Elemental composition and double layer capacitance of nanoengineered cobalt electrocatalysts investigated in this study.

Electrocatalyst	Co ^a^ (wt %)	O ^a^ (wt %)	Cu ^a^ (wt %)	C_dl_ ^b^ (mF/cm^2^)
Cu	0	0	100	-
Co@Cu-10	71.0	18.3	10.7	1.69
Co@Cu-20	74.1	16.1	9.8	3.44
Co@Cu-30	80.0	10.9	9.1	3.91

^a^ Obtained from EDX analysis. ^b^ Electrochemical double layer capacitance.

## Data Availability

The data supporting the conclusions of this article will be made available by the authors upon reasonable request.

## Supplementary Materials

The following supporting information can be downloaded at: https://www.mdpi.com/article/10.3390/nano14110946/s1, Figure S1: Low and high magnification scanning electron microscope images of cobalt films electrodeposited using the dynamic hydrogen bubble templating method: (a) Co@Cu-10, (b) Co@Cu-20, (c) Co@Cu-30 at low magnification and (d) Co@Cu-10, (e) Co@Cu-20, and (f) Co@Cu-30 at high magnification. Figure S2: XPS spectra of Co@Cu 10s: (a) survey spectra, (b) C1s, (c) O1s, and (d) Co 2p. Figure S3: XPS spectra of Co@Cu 20s: (a) survey spectra, (b) C1s, (c) O1s, and (d) Co 2p. Table S1: Elemental composition and percentage of Co@Cu 10s, Co@Cu 20s, and Co@Cu 30s analysed by XPS. Table S2: Comparison of Co based electrocatalyst with our synthesised catalyst. References [65,66,67,68,69,70,71,72] are cited in the Supplementary Materials.
