# Supplementary material for: Nanoengineered Cobalt Electrocatalyst for Alkaline Oxygen Evolution Reaction
Source: Nanomaterials (Basel). 2024 May 28;14(11):946. doi: 10.3390/nano14110946 (PMC11173492; doi:10.3390/nano14110946)
Supplement: Supplementary file 1 [file nanomaterials-14-00946-s001.zip › nanomaterials-3019778-supplementary.pdf]

Nanoengineered Cobalt Electrocatalyst for Alkaline Oxygen Evolution Reaction

Venkatachalam Rajagopal,[a] Sunil Mehla,[a] Lathe A. Jones,[a] and Suresh K. Bhargava.[a,*]

[a]*Centre for Advanced Materials and Industrial Chemistry (CAMIC), School of Science, STEM College, RMIT University, GPO Box 2476, Melbourne, Victoria 3001, Australia.*

Corresponding author: E-mail: suresh.bhargava@rmit.edu.au*

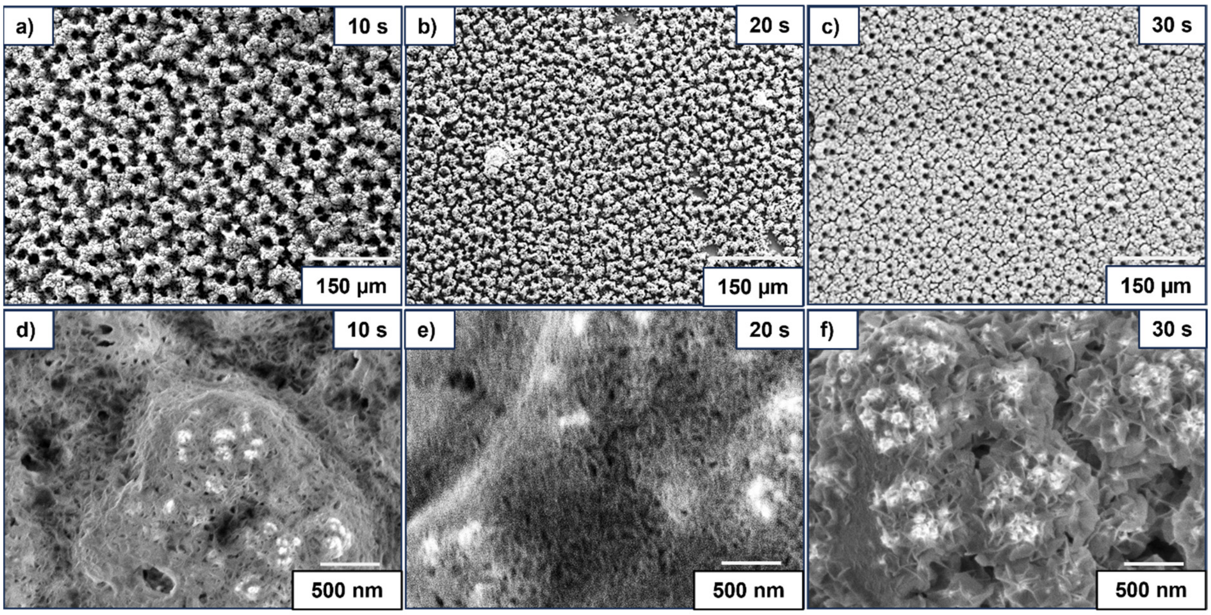

Figure S1 Low and high magnification scanning electron microscope images of cobalt films electrodeposited using the dynamic hydrogen bubble templating method: a) Co@Cu-10, b) Co@Cu-20, c) Co@Cu-30 at low magnification and d) Co@Cu-10, e) Co@Cu-20, and f) Co@Cu-30 at high magnification.

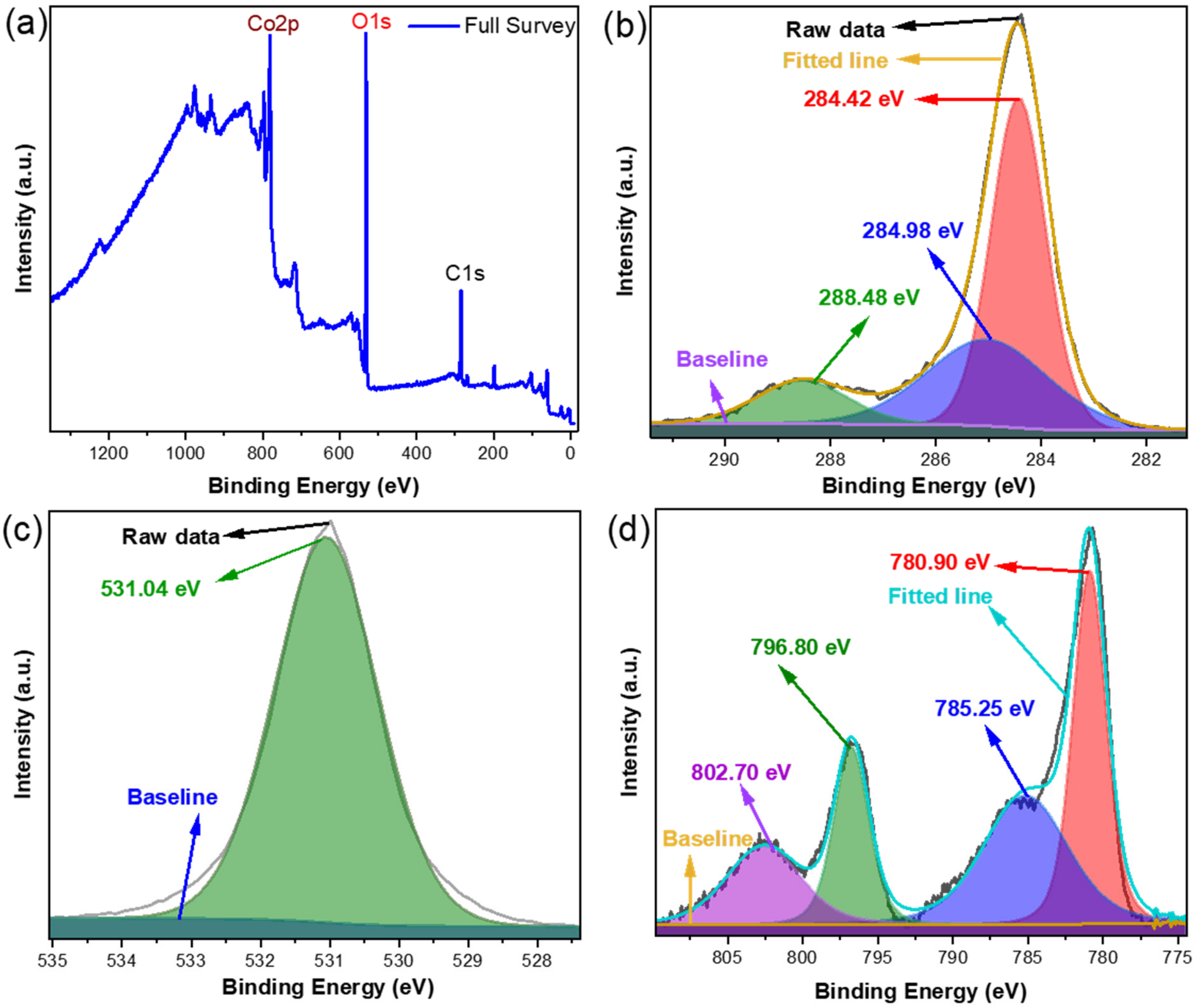

Figure S2 XPS spectra of Co@Cu 10s: (a) survey spectra, (b) C1s, (c) O1s, and (d) Co 2p

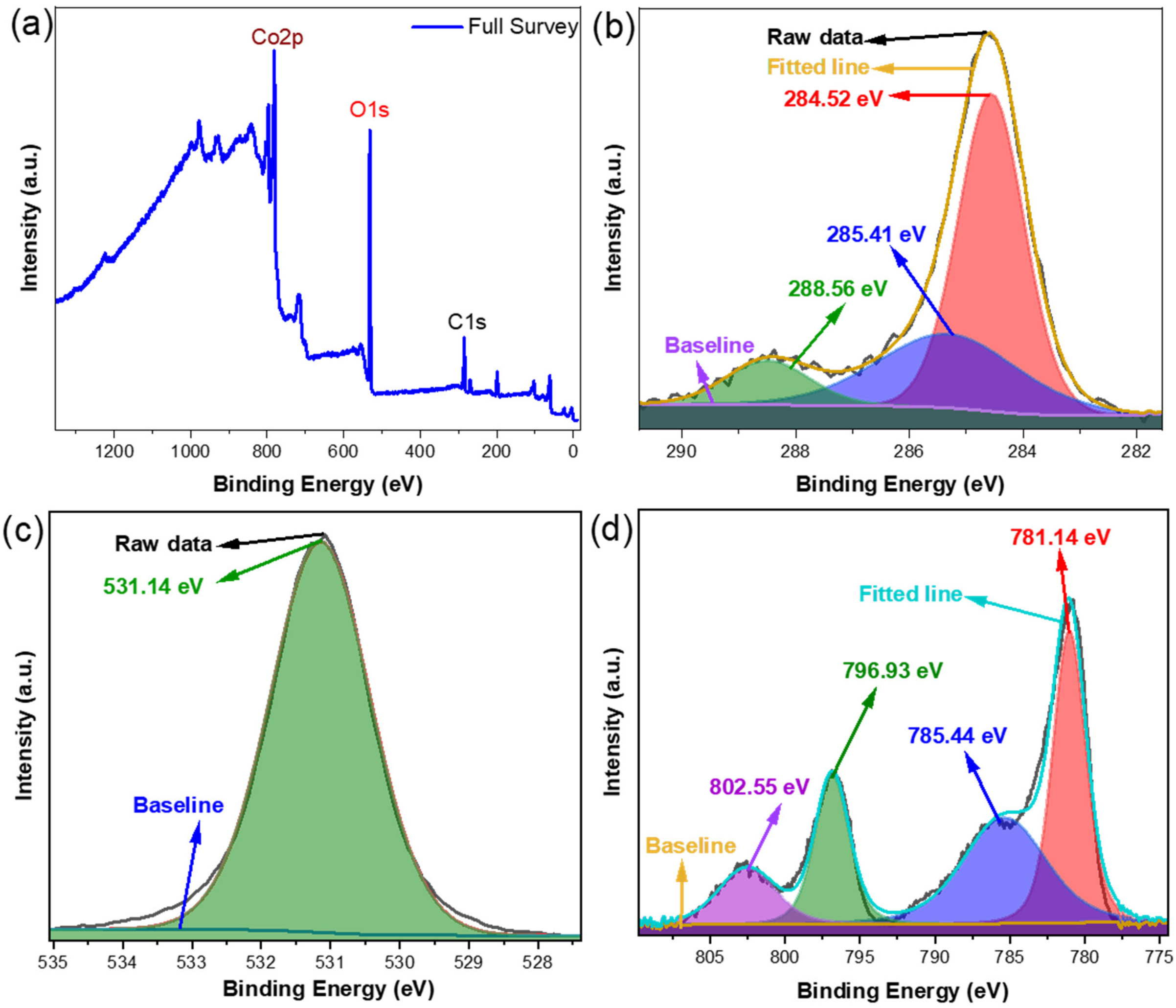

Figure S3 XPS spectra of Co@Cu 20s: (a) survey spectra, (b) C1s, (c) O1s, and (d) Co 2p.

Table S1. Elemental composition and percentage of Co@Cu 10s, Co@Cu 20s, and Co@Cu 30s analysed by XPS.

S. No	Samples	C1s			O1s	Co2p			
		C=C (%)	C-C (%)	C-O-C (%)	Co-OH (%)	Co^{+2} $2p_{3/2}$ (%)	Satellite $2p_{3/2}$ (%)	Co^{+2} $2p_{1/2}$	Satellite $2p_{1/2}$ (%)
1	Co@Cu-10	55.42	32.27	12.30	100	33.73	31.31	17.90	17.06
2	Co@Cu-20	59.83	29.61	10.54	100	35.88	32.65	19.22	12.23
3	Co@Cu-30	60.24	26.67	13.07	100	36.10	31.16	18.74	13.99

Calculation of Turnover frequency (TOF)

$$TOF = \frac{\mathrm{j} \times \mathrm{s}}{\mathrm{nF}}$$

j = Current density

s = Geometrical Surface area (1 cm^2)

n = Number of Electron transfer in OER (4 for OER)

F = Faraday Constant (96485 C/mol)

Table S2. Comparison of Co based electrocatalyst with our synthesised catalyst.

S.No	Catalyst	Synthesis Method	Overpotential (mV)	Current density (mA/cm^2)	Tafel Slope (mV/dec^{-1})	Ref
1	$Co(OH)_2$@Ni	Electrodeposition	330	10	100	1
2	Ni–Co–S	Electrodeposition	363	100	109	2
3	CoP_3	Solid-state reaction method	343	10	76	3
4	CoP	Solid-state reaction method	400	10	57	3
5	CoMoNiS–NF	Hydrothermal	405	10	71	4
6	CoO/$CoSe_2$	Hydrothermal	510	10	137	5
7	Fe_3C–Co/NC	Template-removal method	340	10	-	6
8	CoP_3 NAs	Synthetic method	334	10	62	7
9	Co_3O_4	Electrodeposition	303	100	75.6	8
10	Co@Cu 30s	DHBT Electrodeposition	360	10	37	This work

References

1 A. Roy, M. Z. Tariq, M. La, D. Choi and S. J. Park, *J. Electroanal. Chem.*, 2022, **920**, 116633.

2 T. Liu, X. Sun, A. M. Asiri and Y. He, *Int. J. Hydrogen Energy*, 2016, **41**, 7264–7269.

3 T. Wu, M. Pi, X. Wang, D. Zhang and S. Chen, *Phys. Chem. Chem. Phys.*, 2017, **19**, 2104–2110.

4 Y. Yang, H. Yao, Z. Yu, S. M. Islam, H. He, M. Yuan, Y. Yue, K. Xu, W. Hao, G. Sun, H. Li, S. Ma, P. Zapol and M. G. Kanatzidis, *J. Am. Chem. Soc.*, 2019, **141**, 10417–10430.

5 K. Li, J. Zhang, R. Wu, Y. Yu and B. Zhang, *Adv. Sci.*, 2015, **3**, 1–7.

6 C. C. Yang, S. F. Zai, Y. T. Zhou, L. Du and Q. Jiang, *Adv. Funct. Mater.*, 2019, **29**, 1–12.

7 T. Wu, M. Pi, D. Zhang and S. Chen, *J. Mater. Chem. A*, 2016, **4**, 14539–14544.

8 W. Gu, L. Hu, X. Zhu, C. Shang, J. Li and E. Wang, *Chem. Commun.*, 2018, **54**, 12698–12701.
